# Point Cloud Completion of Plant Leaves under Occlusion Conditions Based on Deep Learning

**DOI:** 10.34133/plantphenomics.0117

**Published:** 2023-11-15

**Authors:** Haibo Chen, Shengbo Liu, Congyue Wang, Chaofeng Wang, Kangye Gong, Yuanhong Li, Yubin Lan

**Affiliations:** ^1^ Experimental Basis and Practical Training Center, South China Agricultural University, Guangzhou, China.; ^2^ National Center for International Collaboration Research on Precision Agricultural Aviation Pesticides Spraying Technology, Guangzhou, China.; ^3^ College of Electronics Engineering (College of Artificial Intelligence), South China Agricultural University, Guangzhou, China.; ^4^ College of Engineering, South China Agricultural University, Guangzhou, China.; ^5^ Department of Biological and Agricultural Engineering, Texas A&M University, College Station, TX, United States.

## Abstract

The utilization of 3-dimensional point cloud technology for non-invasive measurement of plant phenotypic parameters can furnish important data for plant breeding, agricultural production, and diverse research applications. Nevertheless, the utilization of depth sensors and other tools for capturing plant point clouds often results in missing and incomplete data due to the limitations of 2.5D imaging features and leaf occlusion. This drawback obstructed the accurate extraction of phenotypic parameters. Hence, this study presented a solution for incomplete flowering Chinese Cabbage point clouds using Point Fractal Network-based techniques. The study performed experiments on flowering Chinese Cabbage by constructing a point cloud dataset of their leaves and training the network. The findings demonstrated that our network is stable and robust, as it can effectively complete diverse leaf point cloud morphologies, missing ratios, and multi-missing scenarios. A novel framework is presented for 3D plant reconstruction using a single-view RGB-D (Red, Green, Blue and Depth) image. This method leveraged deep learning to complete localized incomplete leaf point clouds acquired by RGB-D cameras under occlusion conditions. Additionally, the extracted leaf area parameters, based on triangular mesh, were compared with the measured values. The outcomes revealed that prior to the point cloud completion, the *R*^2^ value of the flowering Chinese Cabbage’s estimated leaf area (in comparison to the standard reference value) was 0.9162. The root mean square error (RMSE) was 15.88 cm^2^, and the average relative error was 22.11%. However, post-completion, the estimated value of leaf area witnessed a significant improvement, with an *R*^2^ of 0.9637, an RMSE of 6.79 cm^2^, and average relative error of 8.82%. The accuracy of estimating the phenotypic parameters has been enhanced significantly, enabling efficient retrieval of such parameters. This development offers a fresh perspective for non-destructive identification of plant phenotypes.

## Introduction

Plant phenotyping research is currently focused on utilizing 3-dimensional (3D) point cloud technology for non-invasive measurement of plant phenotypic parameters [1–3]. The utilization of 3D point cloud technology in plant phenotyping not only eliminates the drawbacks of traditional artificial measurement methods that can potentially harm the plants but also eradicates any errors caused by the loss of spatial information in the 2-dimensional image approach. Passive 3D reconstruction techniques, like structure from motion (SFM) and multi-view stereo (MVS) algorithms, are commonly used in the field of plant phenotyping. This method requires only a camera to capture multi-view pictures of the plant, enabling relatively complete point cloud data to be generated. The process is simple to operate. The use of passive 3D reconstruction techniques for plant phenotypic parameter extraction has been widely proven to be effective [4–6], but such methods involve high computational costs, and dense reconstruction can take a significant amount of time. In recent years, active 3D reconstruction methods employing 3D sensors such as laser radars, binocular cameras, and time of flight (ToF)-based depth cameras have been extensively employed for plant phenotypic information extraction [7–9].

The use of 3D sensors for plant point cloud generation creates 2.5D images and is influenced by occlusions between plants, leading to incomplete data acquisition. This condition hinders the accurate extraction of phenotypic parameters. The accurate measurement of phenotypic parameters relies heavily on the acquisition of complete plant point cloud data. To address the challenge of incomplete data, researchers have focused on the development of point cloud completion methods in graphics. Registration methods, geometry-based methods, and database-based methods [10] are among the existing techniques employed for this purpose.

To achieve better 3D reconstruction, some researchers have employed splicing techniques that involve combining multi-view point clouds obtained via 3D sensors. These techniques leverage the point cloud features of overlapping areas or the registration method based on the calibrator to attain complementarity between the various views [11,12]. Most of the registration algorithms currently available are designed for rigid point clouds, making them unsuitable for flexible plants subjected to environmental factors such as wind in outdoor settings or shaking in indoor environments. These factors can cause shape transformation, leading to registration errors that subsequently affect the accuracy of phenotypic parameter extraction.

Gené-Mola et al. [13] utilized shape fitting technology to estimate the size of fruits by fitting point clouds of incomplete spherical fruits (apples) with varying occlusion rates. Luo et al. [14] employed Kinect V2 to capture the point cloud of lettuce leaves from a bird’s eye view, and utilized the geometric relationship of the leaves’ shape to complete the occluded leaf point cloud. They further extracted various parameters from the completed point cloud, including the leaf area. Nonetheless, this type of approach heavily relies on the shape and geometric properties of the studied object, making it unsuitable for complicated scenarios.

The introduction of PointNet [15] has sparked the integration of deep learning into point cloud analysis. Many researchers have explored how to employ deep learning techniques to enhance point cloud completion, examples of which include PCN [16], VRCNet [17], and ShapeInversion [18]. Muhammad et al. [19] proposed Reinforcement Learning Agent Controlled GAN Network (RL-GAN-NET), which takes an incomplete point cloud as input and generates a completed point cloud with increased realism by addressing the noise-containing regions. Wang et al. [20] proposed a progressive upsampling network, which aims at transforming a sparse point cloud into a denser one. Point Fractal Network (PF-Net) proposed by Huang et al. [21] takes the incomplete point cloud as the input of the network. By preserving the spatial layout of the original incomplete point cloud, the model predicts the geometry of the missing areas, which can maximize the retention of the point cloud information obtained from the sensor.

Li et al. [22] introduced a plant leaf point cloud completion approach that used a depth coder–decoder framework. The encoder processes the incomplete point cloud of the plant leaf as a vector of shape features, and the decoder is trained on these data to predict the complete point cloud of the leaf. To complete the single leaf point cloud, Xiao et al. [23] utilized a multi-scale feature extraction module and a point cloud pyramid decoder to complete a single leaf point cloud obtained from Kinect v2. However, their method produced a complete point cloud as the output, altering the original point cloud data obtained by the sensor. Zeng et al. [24] introduced a multi-scale geometry-aware Transformer network for point cloud completion of plant seedlings, which presented a novel approach. However, their method directly completed the entire plant, which led to the loss of some localized features.

This study explored the application of neural network-based point cloud completion to achieve 3D reconstruction of plants under occlusion. Taking flowering Chinese Cabbage leaves as the research object, which exhibit complex structures with large areas, numerous folds, and bends that pose considerable challenges for 3D reconstruction. Firstly, the point cloud dataset of cabbage leaves was created. The PF-Net algorithm was then utilized to train the dataset, and an incomplete point cloud of the leaves was obtained using an Azure Kinect camera from a single viewpoint. This incomplete point cloud was supplemented by the neural network to achieve full 3D reconstruction. Subsequently, the reconstructed point cloud was compared with the MVS-SFM algorithm to validate the reconstruction accuracy. Finally, leaf area parameters were extracted from the reconstructed leaf point cloud to validate the effectiveness of the approach.

## Materials and Methods

This study took “Youlv 501 caixin” [25] as the research object, a leafy vegetable commonly used in Chinese cuisine, also known as Chinese flowering cabbage, hereinafter referred to as Chinese Cabbage. First, the leaf point cloud dataset was created utilizing the MVS-SFM algorithm and Azure Kinect camera respectively to train the neural network. The raw plant point cloud was obtained from the top view through the Azure Kinect camera. Preprocessing techniques of point cloud including straight-through filtering, color threshold filtering, and statistical filtering were carried out first to extract the target plant point cloud. The region growing segmentation algorithm [26] was used to segment the single leaf point cloud in this study.

**Fig. 1.**
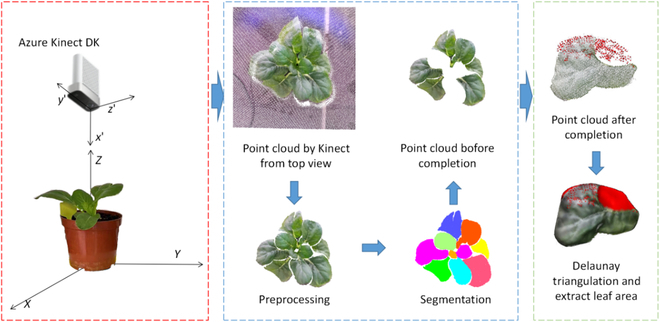
The workflow of the completion of plant point cloud.

Afterward, the completion of the leaf point cloud was carried out by the neural network. After completing the point cloud, the Delaunay 2.5D triangulation algorithm was used to reconstruct the surface of the completed leaf point cloud, and the leaf area S was approximately measured based on the leaf triangulation grid, so as to analyze the robustness and effectiveness of this method. The overall workflow of the method is shown in Fig. 1.

### Data acquisition and preprocessing

Azure Kinect is a ToF-based depth sensor launched by Microsoft, boasting exceptional precision, resolution, and robust resistance to interference. In this study, the color camera functioned in RGBA mode and had a pixel resolution of 1,920 × 1,080. Depth information utilized WFOV (wide field of view) uncompressed mode with a resolution of 1,024 × 1,024 pixels, operating at a frame rate of 15 frames/s within a range of 0.25 to 2.21 m. To ensure that the entire Chinese Cabbage is within the imaging range of the camera and to minimize the effects of the phenomenon where point clouds are denser in close range and sparser in far range when using an RGB-D camera, tests were conducted, and the distance for placing the Azure Kinect was determined to be 0.5 m above the Chinese Cabbage.

Data collection and processing used a graphics workstation equipped with an Intel core i5 10400 processor, 32 GB memory, and an NVIDIA GeForce RTX 3060 12 G graphics card. In the Visual Studio 2019 programming environment, the Azure Kinect development kit, Point Cloud Library [27], OpenCV library, and Open3D library [28] were used to realize point cloud data collection and data processing.

By analyzing the XYZ data and RGB data of the point cloud, the point cloud of Chinese Cabbage in the region of interest was separated from the background point cloud as well as the point cloud of flower pots and soil using straight-through filtering and color threshold filtering. The statistical filtering algorithm was further used to remove the outlier noise, and the pre-processed point cloud is shown in Fig. 1, which shows that the point cloud of Chinese Cabbage has been effectively extracted. The leaf point cloud was segmented using the region growth algorithm, and the point cloud that cannot be segmented correctly is manually adjusted on CloudCompare software. Incomplete leaf point clouds were selected and subsequently completed using deep learning.

### Dataset construction

In this study, all of the dataset samples were obtained from real Chinese Cabbage. The complete plant point cloud was obtained by utilizing the MVS-SFM algorithm for the cabbage’s 3D reconstruction, then the original point cloud was subject to noise reduction processing. A total of 150 complete leaf point clouds were obtained through manual segmentation.

A unit spherical bounding box is created for every complete leaf point cloud. The surface of the sphere was equipped with 21 viewpoints, which simulate diversified positions in the authentic scene. To generate the incomplete point cloud, the points far away from the viewpoint were eliminated with 3 different missing ratios of 20%, 35%, and 50%. Eventually, point cloud sampling was conducted using the iterative farthest point sampling (IFPS) [27] to generate input data consisting of 2,048 points. From the deleted farthest points, 512 points were sampled by the IFPS algorithm, and these serve as the ground truth to compute the loss function. Using the aforementioned dataset construction method, a total of 9,450 samples were obtained. Each sample consists of an incomplete point cloud to be completed (2,048 points) along with its corresponding missing ground truth (512 points). These samples were utilized as pre-training inputs for the model.

As illustrated in Fig. 1, the framework of this paper primarily focuses on the completion of incomplete point clouds acquired by a depth camera from a single perspective. Due to the disparate principles underlying the acquisition of point clouds by depth cameras and the MVS-SFM algorithm, the former generates point clouds on a per-pixel basis, resulting in a more uniformly distributed point cloud, whereas the latter primarily derives point clouds from the feature points of images, leading to a non-uniform distribution of the acquired point cloud.

In order to ensure that the trained model is applicable to incomplete point clouds acquired by depth cameras, the Azure Kinect camera was utilized to capture 80 complete leaf point clouds. By applying the same data construction method, a total of 5,040 samples were obtained. These samples were then used to do transfer learning for the pre-trained model, aiming to make the trained parameters more suitable for completing the incomplete leaf point clouds captured by the RGB-D camera, while also addressing the issue of dataset insufficiency.

### Point cloud completion network

**Fig. 2.**
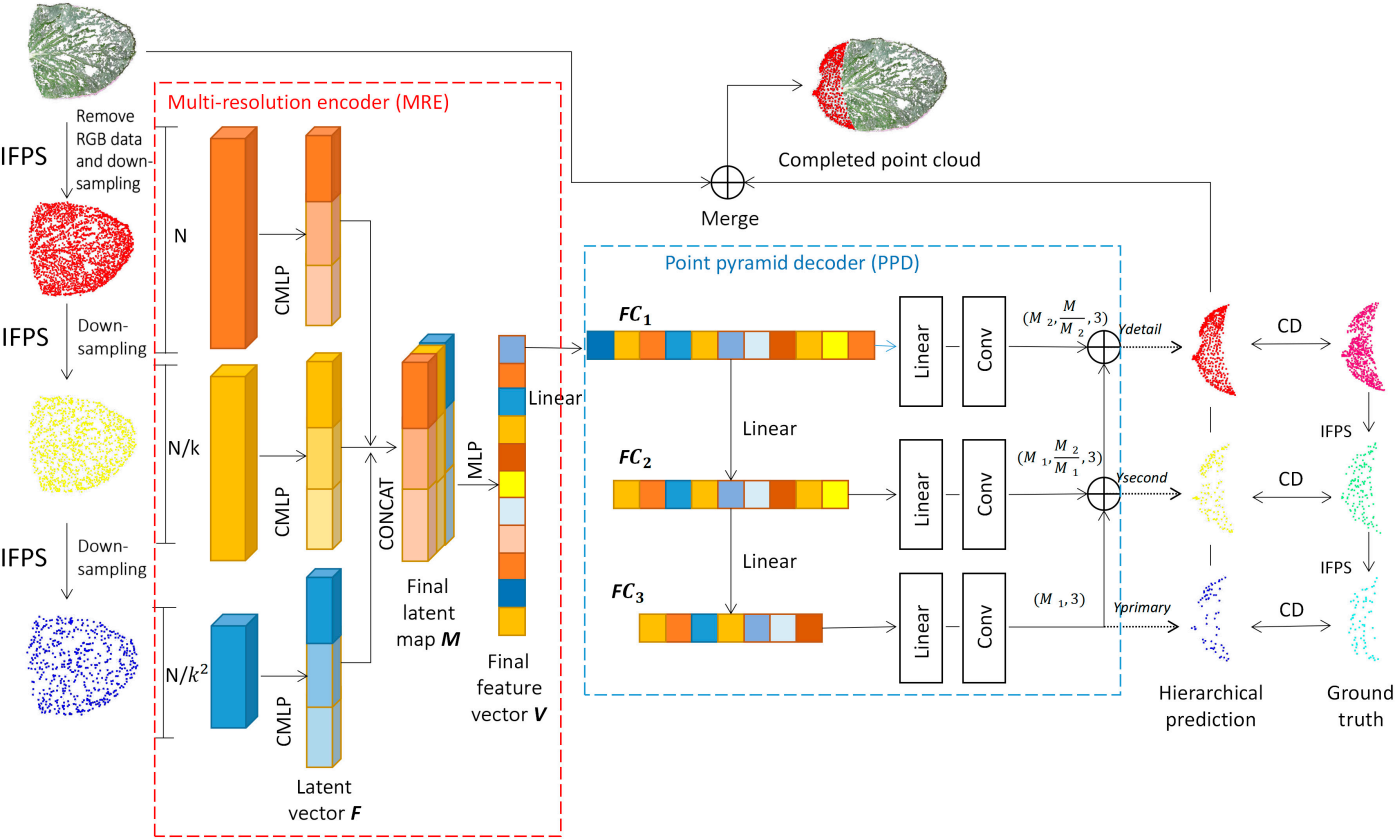
PF-Net architecture for flowering Chinese Cabbage’s leaf point cloud completion.

In this study, PF-Net was used to complete the leaf point cloud, and its network structure is shown in Fig. 2. The initial incomplete leaf point cloud undergoes a processing step to eliminate the RGB data, as the current point cloud deep learning only focuses on the XYZ information of the point cloud. Subsequently, the IFPS algorithm is utilized to perform multi-scale downsampling. The multi-scale feature extraction module (multi-resolution encoder [MRE]) is utilized to extract point cloud features, which are then fused from various depths. The point cloud pyramid decoder (PPD) module is subsequently used to predict multi-scale point cloud generation, ultimately accomplishing the task of completing the leaf point cloud. During the training process, the model is trained using the chamfer distance (CD) as the loss function, which involves calculating the difference between the predicted point cloud and the ground truth point cloud at various scales. The ground truth point clouds at different scales are obtained using the IFPS algorithm.

#### Multi scale feature extraction module

**Fig. 3.**
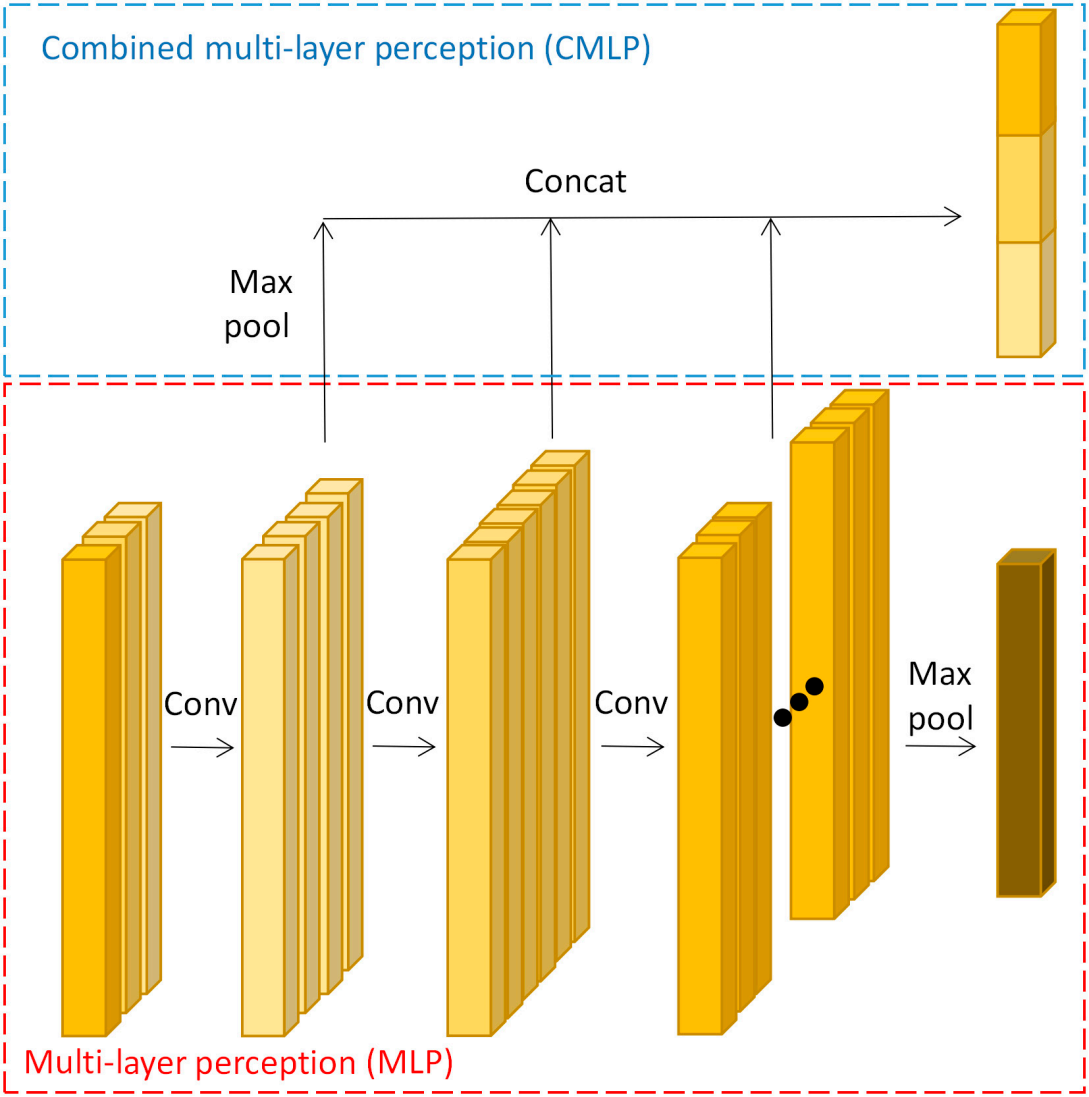
Combined multi-layer perception.

Firstly, the point cloud data are downsampled to varying scales (2,048, 1,024, and 512 points) using the IFPS algorithm. This approach is more efficient at extracting the point cloud skeleton and accurately capturing the point cloud distribution than random sampling methods. As shown in Fig. 2, the MRE module employs the combined multi-layer perception (CMLP) algorithm to extract features from input point clouds of various scales. Figure 3 shows how CMLP connects features that are multidimensional. By simultaneously considering features at various levels, this method incorporates more low-level and high-level feature data, better than MLP that only outputs the last level of features. After obtaining features with 3 different resolutions through CMLP, they are merged together and subsequently input into an MLP to generate the final vector (V).

#### Point cloud pyramid decoder

The completion of the leaf point cloud is achieved by the PPD module illustrated in Fig. 2. The final vector V obtained by the MRE module is outputted by the multilayer perceptron with 3 different resolutions of 512 × 3, 128 × 3 and 64 × 3, and the point clouds generated are gradually changed from rough to fine after the complementary output of 3 different resolutions.

#### Loss function

There are 2 commonly used methods for measuring the distance between 2 point clouds, namely, chamfer distance (CD) and earth mover’s distance (EMD) [29]. The similarity of the 2 point clouds is higher when the values of these measures are lower.

This research has selected the CD as the loss function, as it is more computationally efficient to calculate than EMD. The calculation formula for this measure is as follows:

$$d_{\mathrm{cd}}\left(S_{1},S_{2}\right)=\sum_{x\in S_{1}}‍{\text{min}}_{y\in S_{2}}{\left\|x-y\right\|}_{2}^{2}+\sum_{y\in S_{2}}‍{\text{min}}_{x\in S_{1}}{\left\|x-y\right\|}_{2}^{2}$$ (1)

*d_cd_* is the CD, which represents the mean nearest square distance between the predicted missing point cloud *S*_1_ and the real missing point cloud *S*_2_. Since the point cloud pyramid decoder in the network will output 3 distinct levels of point clouds, the calculation of the multi-stage complementary loss is carried out as follows:

$${\text{Loss}}_{1}=d_{\mathrm{cd}}\left(Y_{1},Y_{\mathrm{gt}}\right)+\alpha d_{\mathrm{cd}}\left(Y_{2},Y_{\mathrm{gt}}^{2}\right)+\beta d_{\mathrm{cd}}\left(Y_{3},Y_{\mathrm{gt}}^{3}\right)$$ (2)

In the formula, *Y*_1_, *Y*_2_, and *Y*_3_ refer to the 3 different level point clouds generated by the point cloud pyramid decoder, while *Y_gt_* represents the actual missing point cloud. Additionally, $Y_{\mathrm{gt}}^{2}$ and $Y_{\mathrm{gt}}^{3}$ are obtained from *Y_gt_* through the IFPS algorithm. The hyperparameters *α* and *β* are also used for the calculation of the multi-stage complementary loss.

### Model training

The model training was conducted on the graphic workstation described in the “Data acquisition and preprocessing” section. The software platform included Windows 10 as the operating system, CUDA version 11.4, Python version 3.7, and PyTorch version 1.9.

In this experiment, the dataset was partitioned into a training set and a validation set with a ratio of 7:3, and a 2-stage training mode was used to compensate for the insufficient dataset. As described in the “Dataset construction” section, the 2-stage training utilized 2 point cloud datasets that were obtained through 2 different methods—one was acquired using the MVS-SFM algorithm, while the other was captured using the Azure Kinect camera.

In the first stage, the leaf point cloud data collected via MVS-SFM algorithm were trained with a batch size of 24 and a learning rate of 1 × 10^−4^. The entire model was trained for 100 epochs. In this study, the hyperparameters *α* and *β* were set as follows: for the first 30 epochs, *α* was 0.01 and *β* was 0.02. From epoch 30 to 80, *α* was adjusted to 0.05 and *β* was adjusted to 0.1, and from epoch 80 to 100, they were fine-tuned to 0.1 and 0.2, respectively. The training process progresses from shallow to deep, gradually increasing the weighting of the level of detail in the multi-scale feature.

For the second stage, the outcomes obtained from the previous stage were utilized via transfer learning to further train the point cloud dataset gathered via the Azure Kinect camera. This was done in order to adapt the model to effectively supplement the leaf point cloud data captured by the depth camera. The parameters used in the second stage of training remained consistent with those employed in the first stage.

**Fig. 4.**
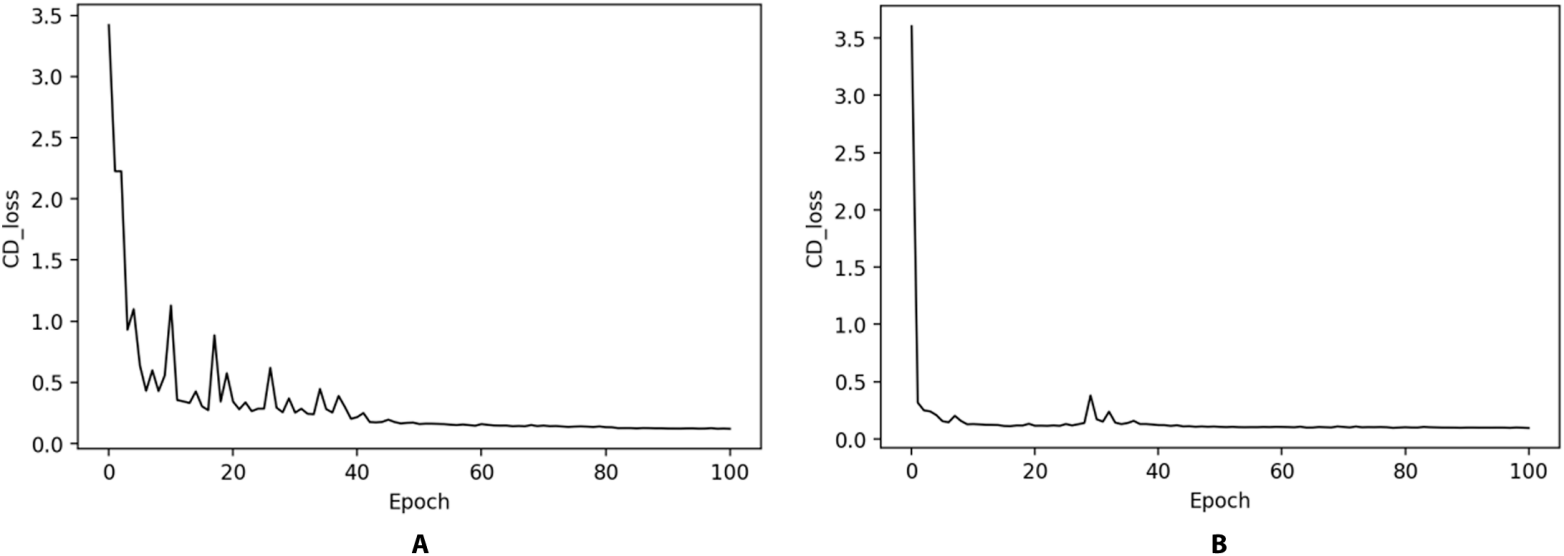
Loss value curves. (A) The decreasing trend of CD values during the first stage of training. (B) The decreasing trend of CD values during the second stage (Transfer Learning).

The weights files were saved along with the loss values of both the training and validation sets. Figure 4A illustrates the decreasing trend of CD values during the first stage of training, while Fig. 4B shows the decreasing trend during the second stage. It was evident that after training, the CD distance effectively decreased, indicating that the predicted missing parts of the point cloud progressively approached the ground truth values during the training process. Notably, Fig. 4B demonstrates a rapid decrease in CD values in the beginning of the second stage training, suggesting that the parameters were optimized swiftly during the transfer learning process. This adaptation enabled the model to be finely tuned for the completion of incomplete leaf point clouds captured by the Azure Kinect.

### The evaluation of reconstruction precision

To evaluate the effectiveness of the proposed 3D reconstruction method in this study, As presented in the methodology flowchart in Fig. 1, the incomplete point clouds of leaves collected in the natural occlusion case were completed. Firstly, the point cloud underwent a decolorization process since current point cloud deep learning solely focuses on the XYZ data, which enables the network only to learn the point cloud’s spatial structure. Subsequently, the point cloud was normalized, and the center of gravity coordinates (G) and scaling ratio (K) were recorded. Then, the normal vector of the leaf was determined using plane fitting, which was utilized to adjust the leaf’s orientation to a flat surface, facing downward. Afterward, the manual fine-tuning was conducted to complete the attitude correction of the leaf point cloud and record the resulting rotation matrix (R). Finally, the trained model was employed to complete the incomplete leaf point cloud, and the point cloud of the accomplished leaf underwent scaling recovery and attitude recovery via the recorded rotation matrix (R), center of gravity coordinates (G), and scaling ratio (K) to achieve complete 3D reconstruction of the plant.

Additionally, the point cloud reconstructed using the MVS-SFM algorithm was used as a reference group. Hui et al. [4] demonstrated that this method can be effectively used for 3D reconstruction work of single plants and can be effectively used for the extraction of crop phenotypic parameters. Following the guidelines provided in the paper, individual vegetable plants were placed indoors without any interference, and a series of 60 to 80 images were captured around the plant using a digital camera (Canon EOS M50). The image sequence was then imported into the VisualSFM software for 3D reconstruction.

In addition to the CD and EMD mentioned in the “Loss function” section, commonly used metrics for measuring the similarity between point clouds include root mean square error (RMSE) and Hausdorff distance (HD) [30]. In this study, we further employed the Chinese Cabbage point cloud reconstructed by the MVS-SFM algorithm as the reference group. We assessed the effectiveness of our reconstruction method by analyzing the distance distribution and quantifying it using RMSE and HD. A smaller RMSE and HD indicate a higher similarity between point clouds.

Since the point clouds reconstructed by different methods may have varying scales and coordinate systems, this study initially conducted scale recovery on point clouds from different groups. The point clouds obtained using Azure Kinect have precise dimensions, measured in millimeters. Therefore, the reference group point cloud was scaled to the same dimension space through a scaling transformation. Subsequently, a registration process was applied to transform point clouds from different reconstruction schemes into the same coordinate system, ensuring substantial overlap. Based on this, distances from every point in the reconstructed point cloud to the nearest point in the reference group point cloud were calculated. The maximum value among all these distances corresponds to the HD.

**Fig. 5.**
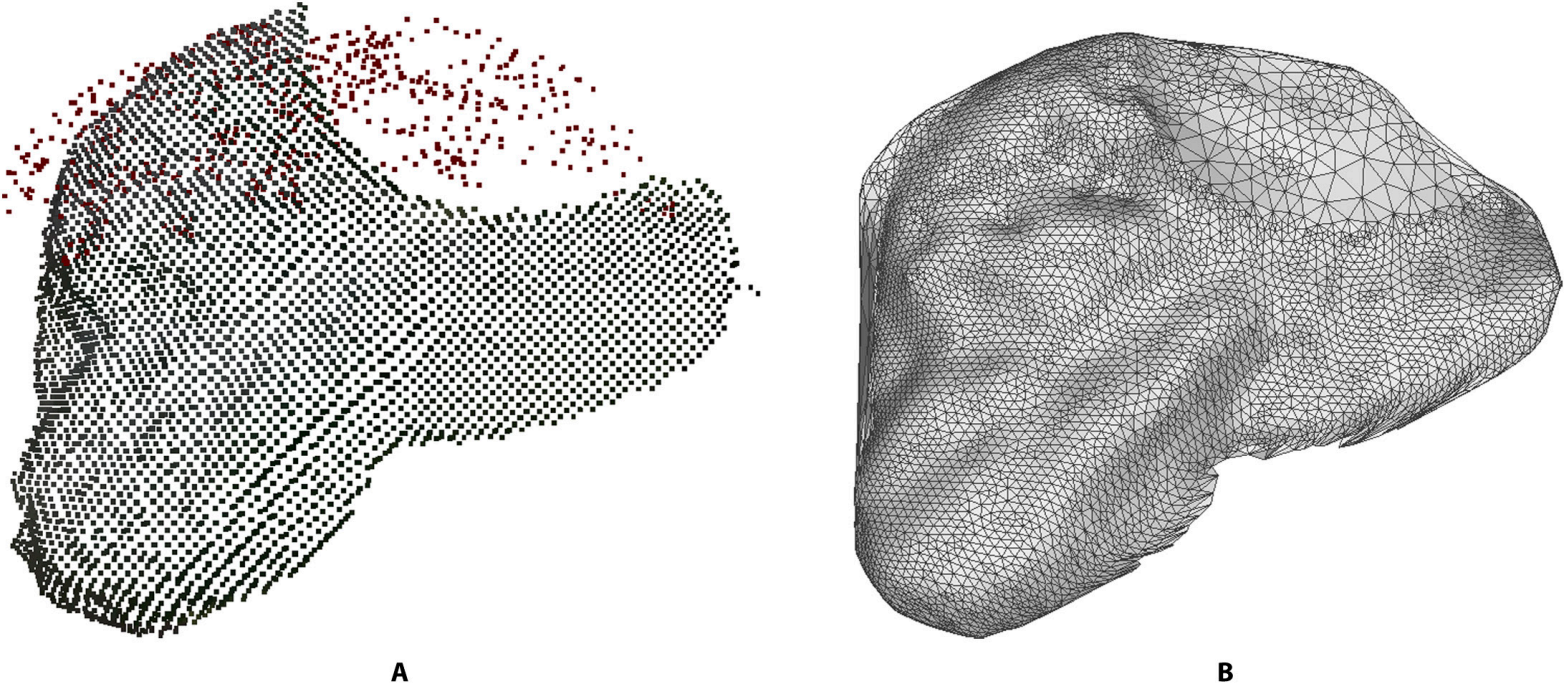
Triangulation of leaf point cloud. (A) The leaf point cloud after completed. (B) The leaf mesh after surface reconstruction.

The ultimate goal of obtaining a complete point cloud is to extract valid phenotypic parameters from it. In order to perform a more comprehensive assessment of the point cloud completion effect achieved, the leaf point cloud was reconstructed into a mesh using the Delaunay 2.5D triangular meshing algorithm, and the resulting mesh was refined by applying Laplace smoothing. Figure 5A displays the completed leaf point cloud, while Fig. 5B showcases the surface reconstruction results. It was evident that through surface reconstruction, the point cloud has been transformed into a mesh. The leaf area was measured based on the leaf triangular mesh approximation, and comparing the results to the reference value obtained by manually measuring the leaf area through WSeen’s LA-S series leaf area meter on the isolated leaf. This was done to confirm the capability of the 3D reconstruction method proposed in this study in extracting crop phenotypic parameters.

## Results

### Analysis of verification set completion results

**Fig. 6.**
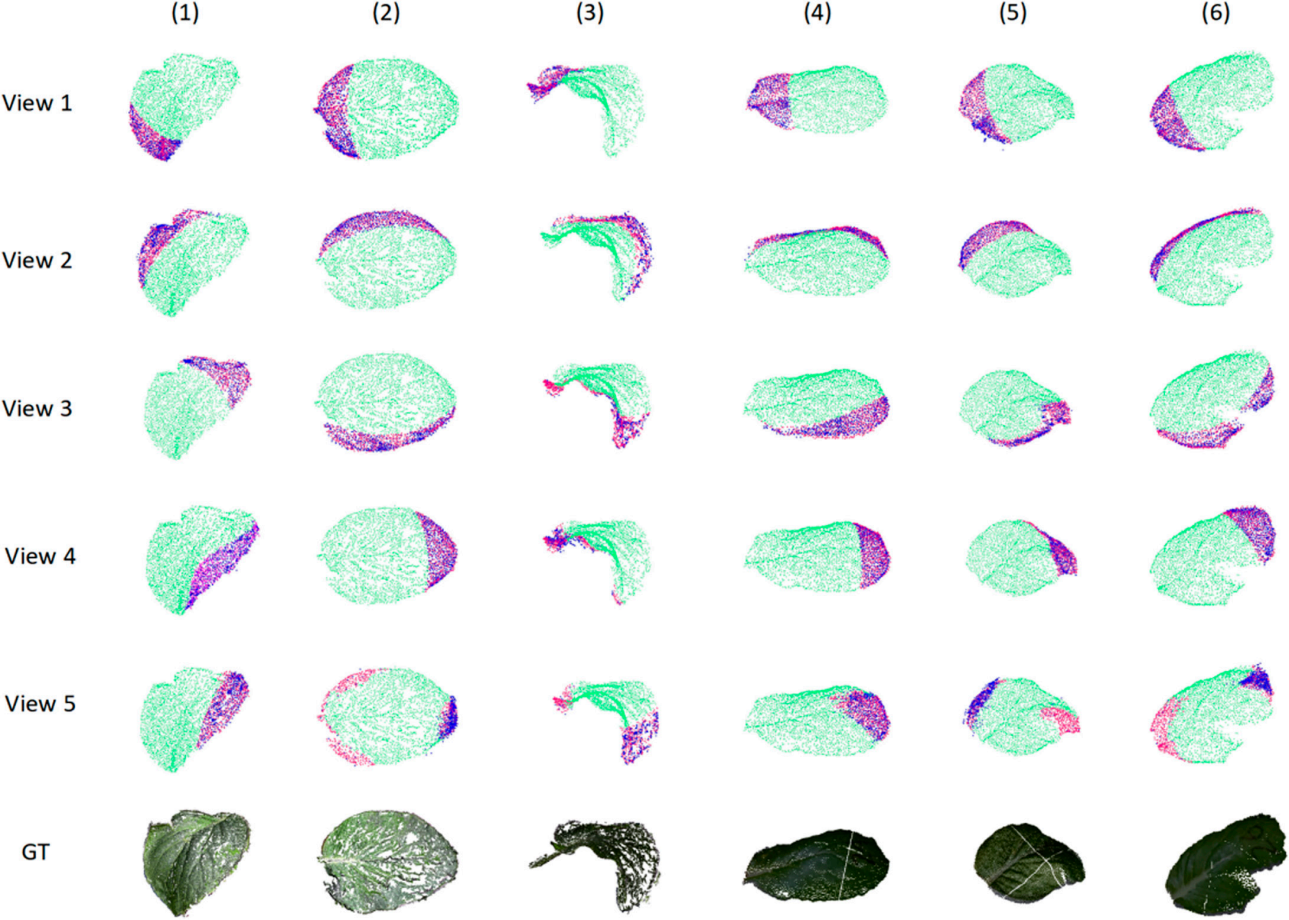
Visualization of the leaf point cloud completion.

To evaluate the efficacy of leaf point cloud completion, several representative leaf point clouds with varying shapes were selected from the verification sets. These point clouds were visualized using MeshLab software [31] to observe the resulting effect. Figure 6 shows the results of the visualization. Groups 1 to 6 display incomplete leaf point clouds with a missing ratio of 20%. Groups 1 to 3 are from the point cloud dataset created using MVS-SFM, while groups 4 to 6 are from the point cloud dataset collected using Azure Kinect. Groups View 1 through View 5 display incomplete point clouds generated through the simulated missing processing from 5 angles and Group GT is the complete leaf point cloud without simulated missing processing. In these visualizations, the green points represent the incomplete point clouds to be completed, pink points represent the real missing point clouds, and blue points represent the predicted complete point clouds by PF-Net.

The results of the visualization demonstrated the successful completion of Chinese Cabbage leaves with varying shapes and bending degrees. Both the point clouds obtained through the MVS-SFM algorithm and collected by Azure Kinect were effectively completed, with a uniform distribution of the resulting point clouds. The point cloud completion results indicated that the network utilized in this paper is effective in completing complex Chinese Cabbage leaves. The completion process successfully completed both the internal and edge points of the leaves, showing a strong ability to complete point clouds. Upon comparing Group View 5, which may have multiple missing areas, with other views, it becomes clear that completion was more effective when the missing areas were concentrated. However, for View5, which has multiple missing areas, the completion effect was poorer, and the resulting point clouds tend to be concentrated on a specific missing area.

**Fig. 7.**
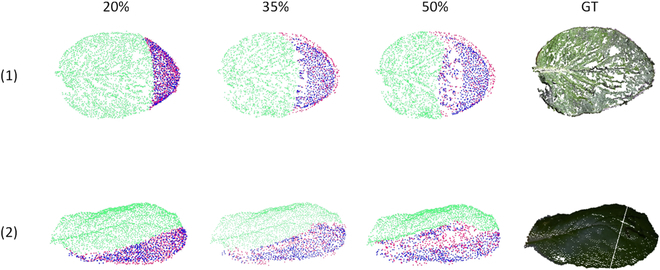
Visualization of the leaf point cloud completion with varying missing ratios.

The completion effect of the leaf point cloud with varying missing ratios is depicted in Fig. 7. In these visualizations, the green points represent the incomplete point clouds to be completed, pink points represent the real missing point clouds, and blue points represent the predicted complete point clouds by PF-Net. It was observable that as the missing ratio increases, the completion effect of the leaf point cloud diminishes. The completion effect of the edge of the leaf point cloud was notably good, but as the missing ratio increases, the missing holes of leaf point cloud became easier to appear, indicating that the network was capable of effectively learning the structural relationships within the target point cloud, which was beneficial for point cloud completion. Any missing holes that may appear can be completed through alternative methods.

### Analysis of completion results under natural occlusion

**Fig. 8.**
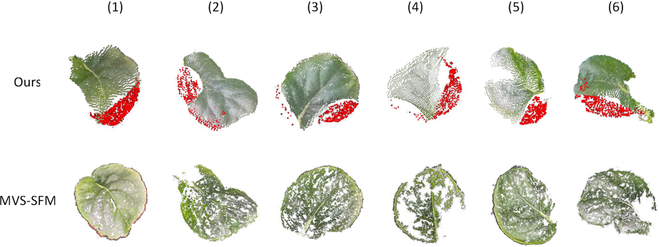
Visualization of the leaf point cloud completion.

**Fig. 9.**
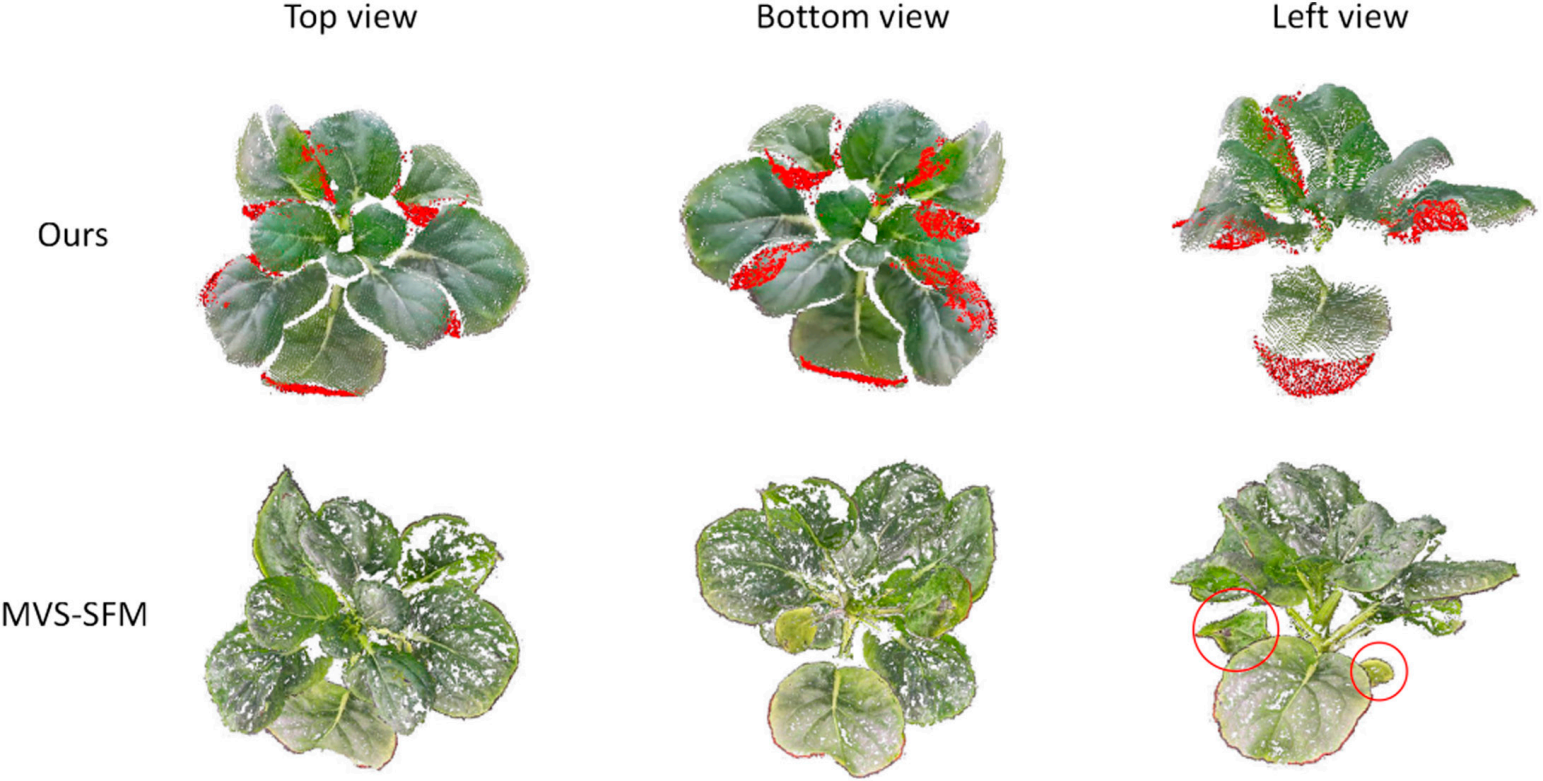
Visualization of the leaf point cloud completion with varying missing ratios.

Figures 8 and 9 show the outcome of single leaf point cloud completion by PF-Net and the 3D reconstruction of the entire plant using the method proposed in this research.

Figure 8 shows a comparative between the effect of completion of incomplete leaf point cloud under natural occlusion conditions and the leaf point cloud reconstructed using the MVS-SFM algorithm. It can be seen that for the densely growing plants, both the reconstruction approach presented in this study and the MVS-SFM algorithm encounter instances where the leaf point cloud was absent. Compared with the point cloud obtained by the MVS-SFM algorithm, the point cloud obtained through Azure Kinect was more uniformly distributed. After the completion process, the point cloud of the leaf became more complete. However, it can also be seen that the completing part of the point cloud was more concentrated, and the completing effect was not optimal when the point cloud of leaf was missing in many places due to multiple occlusions, or the ratio of missing was too high. Pan et al. [17] used multi-view camera rendering to construct the incomplete point cloud dataset that better resembles the incomplete point clouds caused by occlusion during natural conditions.

Figure 9 shows the comparison between the whole plant point cloud reconstructed by the proposed method and the whole plant point cloud reconstructed by the MVS-SFM algorithm. Although the completeness of the plant point cloud was significantly improved after the completion process, it is evident that some leaves that were entirely concealed cannot be reconstructed when compared to the plant reconstructed by the MVS-SFM algorithm. The red circled area in Fig. 9 and the stem portion of the plant are examples of this limitation. Therefore, it was apparent that the plant point cloud reconstructed by the MVS-SFM algorithm was more comprehensive than the one obtained by the proposed method.

### Analysis of reconstruction precision

**Fig. 10.**
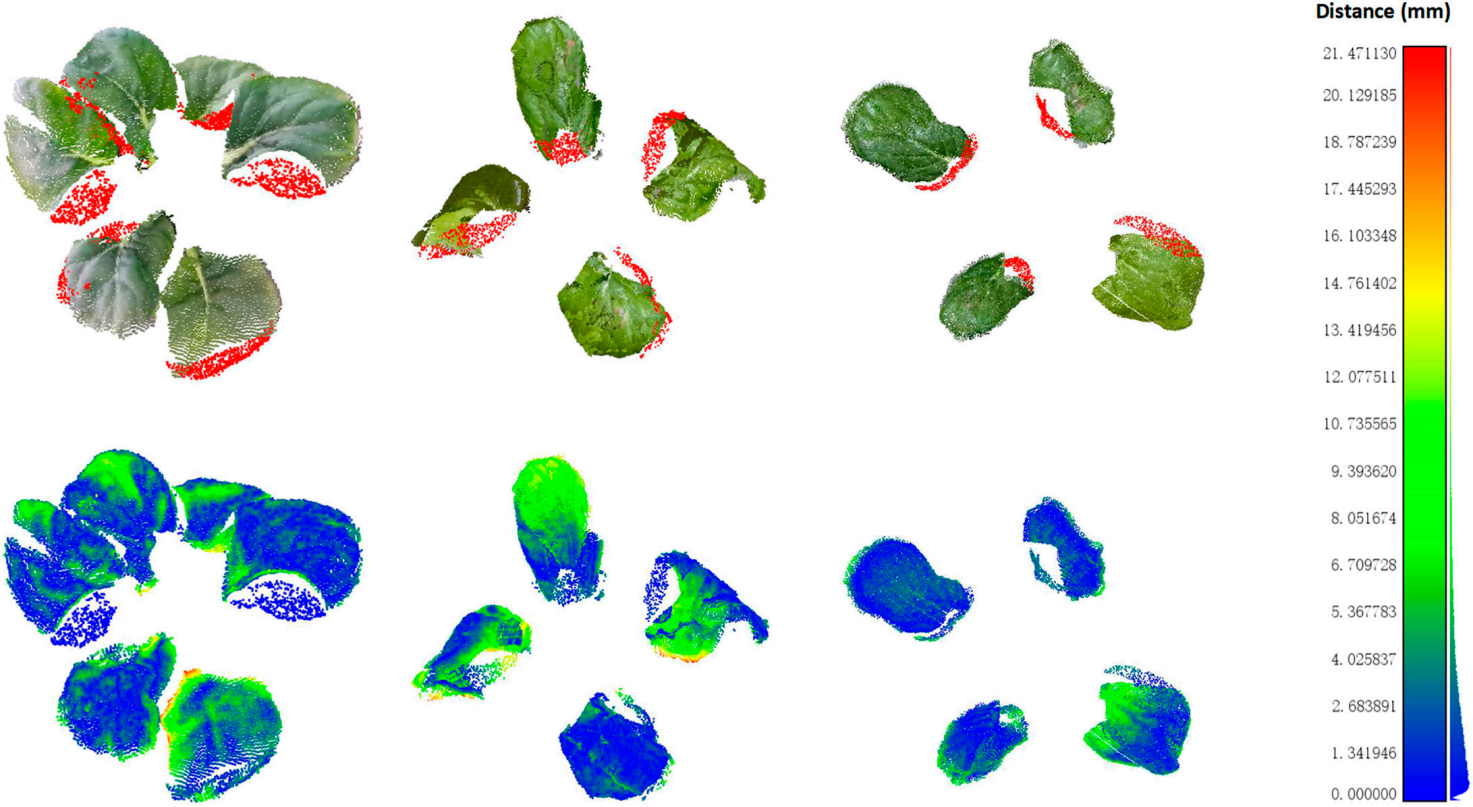
Visualization of distance distribution.

The upper row in Fig. 10 displays the Chinese Cabbage point cloud after completion, while the lower row shows the distance distribution between the partially reconstructed point clouds in this study and the reference group point clouds (exclusively considering the incomplete point cloud), with colors closer to blue indicating greater similarity.

The statistical results demonstrated that about 50% of point pairs have a distance error of less than 2 mm, about 80% of point pairs have a distance error of less than 5 mm, and about 92% have a distance error of less than 8 mm. The RMSE of distance was 3.22 mm, and the HD was 21.40 mm. It can be observed that, in general, the point clouds reconstructed by the 2 methods were moderately consistent. This indicated that the reconstructed point clouds produced by the method proposed in this paper bear a moderate resemblance to those reconstructed by MVS-SFM.

### Analysis of leaf area extraction results

**Fig. 11.**
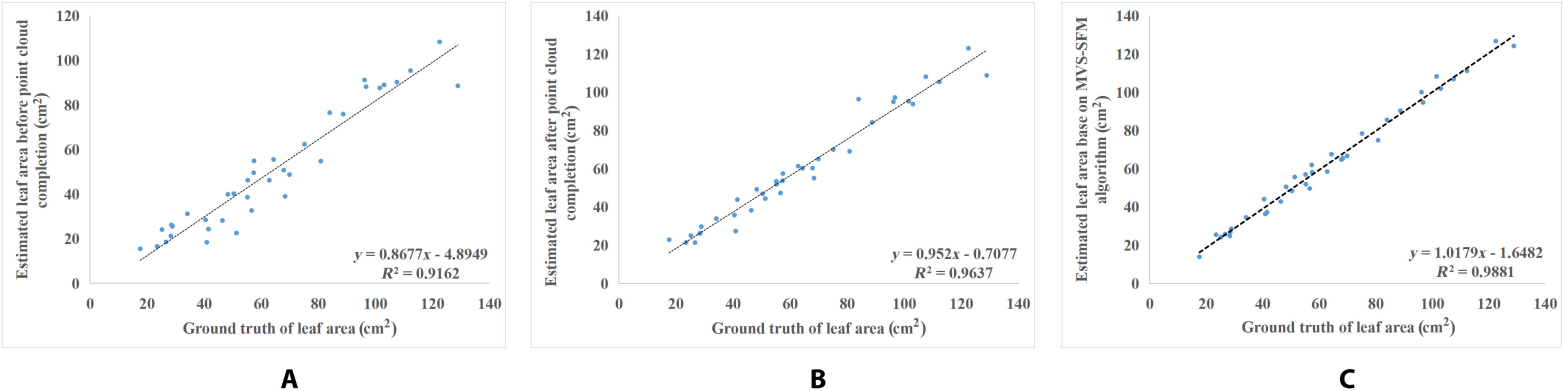
Correlation evaluations of leaf area estimation versus manual measurements. (A) The leaf area estimates before completion. (B) The leaf area estimates after completion. (C) The leaf area estimates for the MVS-SFM group.

This research involved the selection of 38 individual leaf point clouds from 6 pots of Chinese Cabbage. Triangular mesh-based leaf area extraction was utilized, and the effectiveness of this method was quantitatively analyzed by comparing it with manual measurements. Correlation evaluations of leaf area estimation versus manual measurements are depicted in Fig. 11A to C, demonstrating the effects of point cloud after completion on the estimates of leaf area and comparing them with the results obtained using the MVS-SFM reconstructed point clouds.The *R*^2^ of the estimated leaf area before point cloud completion was 0.9162, the RMSE was 15.88 cm^2^, and the average relative error was 22.11%. After the point cloud was completed, the *R*^2^ of the estimated leaf area was 0.9637, the RMSE was 6.79 cm^2^, and the average relative error was 8.82%. For the MVS-SFM reference group, the *R*^2^ of the estimated leaf area was 0.9881, the RMSE was 3.40 cm^2^, and the average relative error was 5.56%. Comparing Fig. 11A and B, it was evident that the accuracy of leaf area estimation has been enhanced after point cloud completion. Comparing Fig. 11B and C, it can be seen that the estimated leaf area of leaf point cloud reconstructed using the method proposed in this study was close to the estimated leaf area of leaf point cloud reconstructed by the MVS-SFM algorithm, showing that it has validity.

## Discussion

The proposed plant 3D reconstruction method, utilizing a depth camera and deep learning point cloud complement, effectively addresses the 2.5D imaging features of depth cameras and leaf occlusion during depth camera imaging, enabling the reconstruction of plants in 3D using only one perspective, providing a new idea for the research of plant phenotype based on active sensors. The precision of the phenotypic parameter estimation has been improved, which verified that the reconstruction method in this study can be used for the extraction of plant phenotypic parameters and has certain accuracy. Nonetheless, certain issues have also been identified, which can be further improved in future work.

1. The model trained in this paper demonstrated good completion performance on the test set, but there was still a certain gap in completing incomplete point clouds collected from the real world. Analysis suggests that the incomplete leaf point cloud dataset in the training samples, which is simulated based on distance sampling, still differs from the actual incomplete leaf point clouds caused by occlusion. In the next step, a more realistic incomplete leaf point cloud dataset will be constructed in a rendering manner to enhance the training quality of the model and improve its generalization ability.

2. In real-world acquisition tasks, due to the uncertainty of target attitude and sensor attitude, the collected point cloud attitude is uncertain. Nevertheless, point cloud data exhibit the essential characteristic of rotation invariance, and the application of point cloud based on deep learning is very sensitive to the attitude of point cloud. Some point cloud applications that utilize deep learning have focused mainly on the ideal situation, disregarding any rotational transformations. For example, some applications assumed that all objects were viewed from a fixed, identical angle [32]. The point cloud orientation in the Shapenet-Part dataset [33] used in PF-Net was consistent, and the trained network can only complete the point cloud for adjusting the correct attitude. Therefore, the point cloud orientation estimation has remained a significant challenge in point cloud processing [34]. This research addressed the challenge of sampling uncertainty in real-world scenarios by employing the plane fitting method to estimate the normal vector of the leaf. We utilized this technique to adjust the leaf point cloud to be horizontal and used manual fine-tuning to complete the leaf point cloud orientation correction, rendering it appropriate for use in the completion network. However, the current method requires manual intervention and lacks full automation. To address this issue, we suggest exploring a multi-stage completion network that is capable of learning the proper pose of the model, adjusting it accordingly, and completing it to tackle the rotation-invariant challenge of point cloud completion tasks.

## Data Availability

PF-Net is available at https://github.com/zztianzz/PF-Net-Point-Fractal-Network, and the repository for this paper is available at https://github.com/newsymbobo/Point-cloud-completion-of-plant-leaves. The data of this study are available from the corresponding author upon request.

## Supplementary Materials

Supplementary 1 Fig. S1. Schematic diagram of principle of incomplete point cloud dataset production. Fig. S2. The challenge of rotation invariance in the task of point cloud segmentation. Fig. S3. The point cloud reconstructed using VisualSFM software. Fig. S4. Standardizing the scale and performing registration to ensure significant overlap. Fig. S5. The measurement of leaf area using WSeen’s LA-S series leaf area meter.
