# Supplementary material for: Point Cloud Completion of Plant Leaves under Occlusion Conditions Based on Deep Learning
Source: Plant Phenomics. 2023 Nov 15;5:0117. doi: 10.34133/plantphenomics.0117 (PMC10795496; doi:10.34133/plantphenomics.0117)
Supplement: Supplementary 1 — Fig. S1. Schematic diagram of principle of incomplete point cloud dataset production. Fig. S2. The challenge of rotation invariance in the task of point cloud segmentation. Fig. S3. The point cloud reconstructed using VisualSFM software. Fig. S4. Standardizing the scale and performing registration to ensure significant overlap. Fig. S5. The measurement of leaf area using WSeen’s LA-S series leaf area meter. [file plantphenomics.0117.f1.docx]

Supplementary Materials

**
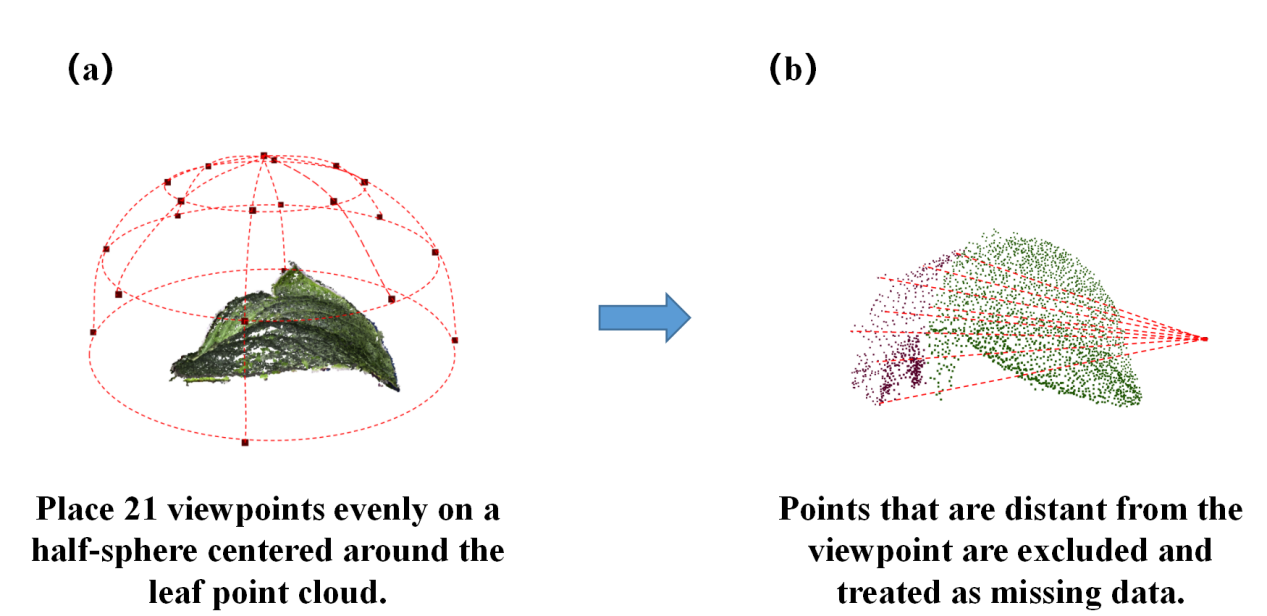
**

**Figure** **S1. Schematic diagram of principle of incomplete point cloud dataset production**


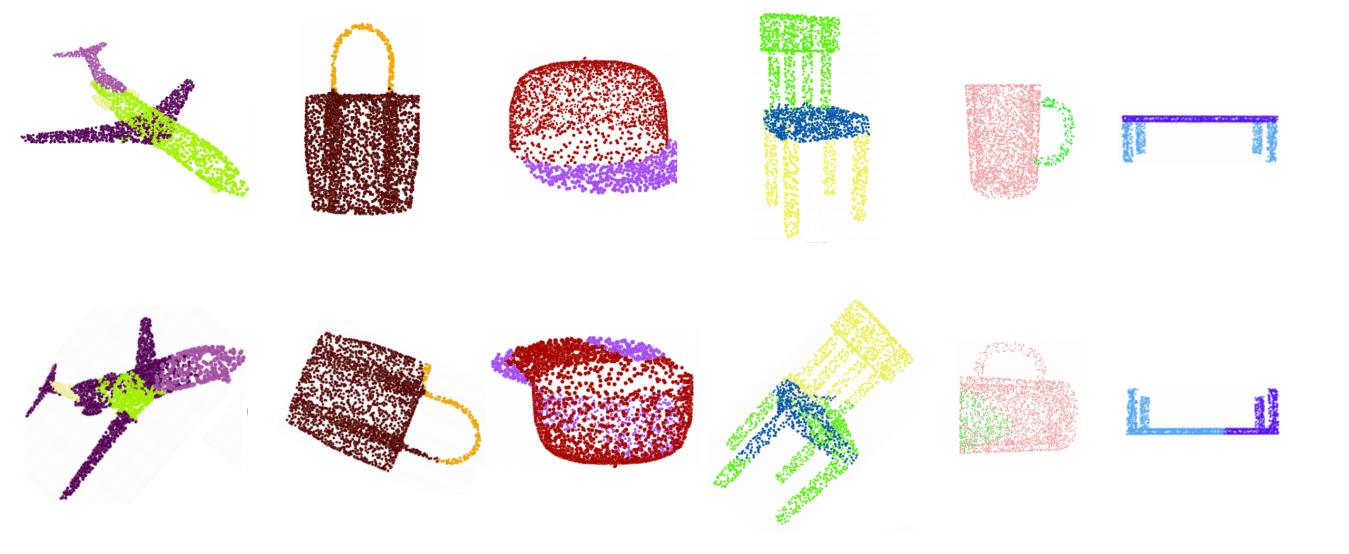


Note:The example in this figure is from the paper "PRIN: Pointwise Rotation-Invariant Network."

**Figure** **S2. The challenge of rotation-invariant in the task of point cloud segmentation.**


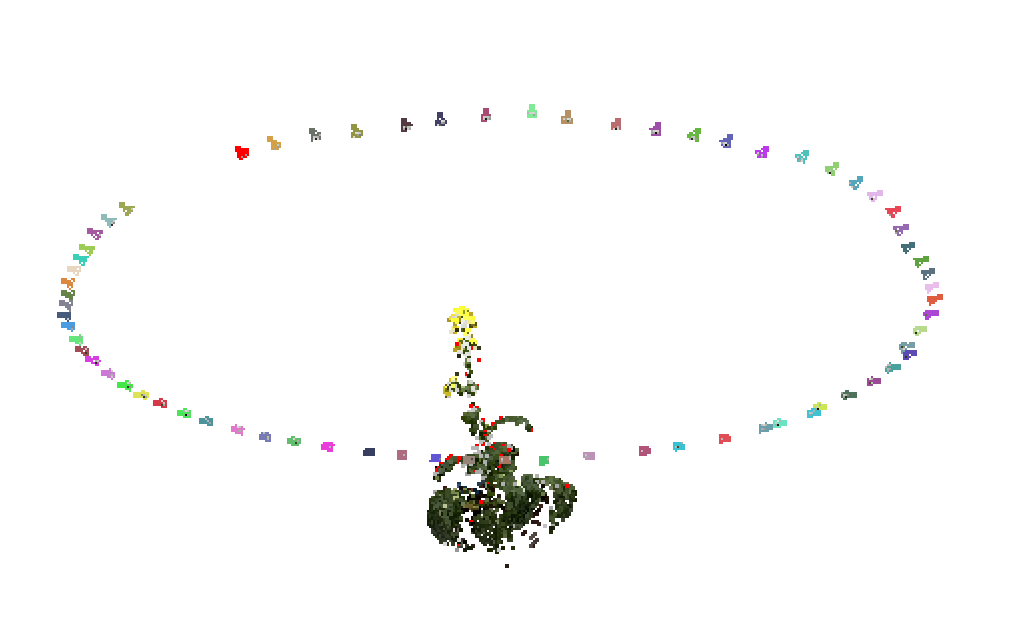

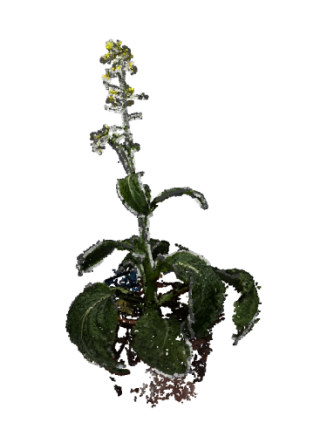


**Figure** **S3. The point cloud reconstructed using VisualSFM software.**

###
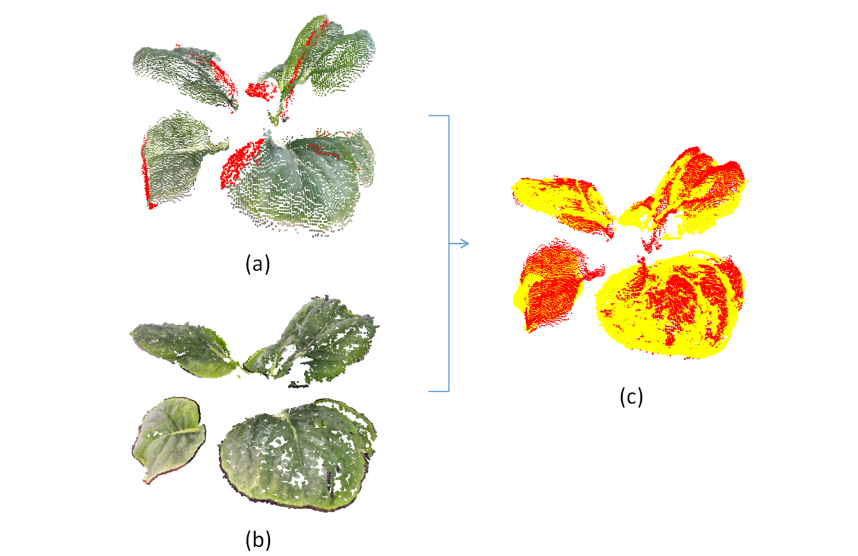


**Figure** **S4. Standardizing the scale and performing registration to ensure significant overlap.**


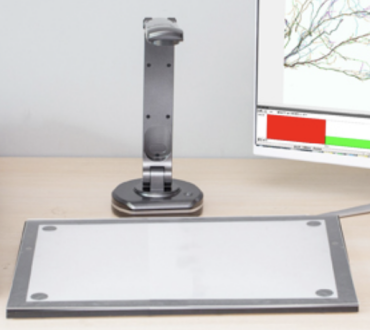

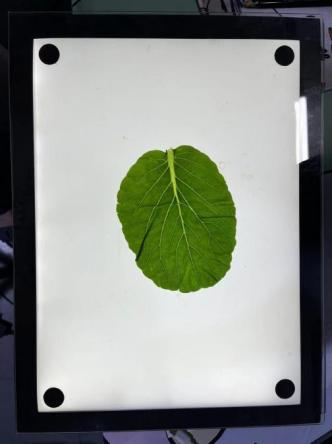


**Figure** **S5. The measurement of leaf area using WSeen's LA-S series leaf area meter.**
